# Small Fiber Neuropathy Associated With the Moderna SARS-CoV-2 Vaccine

**DOI:** 10.7759/cureus.25969

**Published:** 2022-06-15

**Authors:** Farzam Khokhar, Anum Khan, Zaid Hussain, Jianghong Yu

**Affiliations:** 1 Internal Medicine, State University of New York Upstate Medical University, Syracuse, USA; 2 Internal Medicine, New York Institute of Technology College of Osteopathic Medicine, Old Westbury, USA; 3 Internal Medicine, Aga Khan University Hospital, Karachi, PAK; 4 Rheumatology, State University of New York Upstate Medical University, Syracuse, USA

**Keywords:** peripheral sensory neuropathy, moderna mrna vaccine adverse effects, moderna covid-19 vaccine, neurological side effects, covid-19 vaccine, small fiber neuropathy

## Abstract

Efforts of controlling viral transmission began soon after the first cases of coronavirus disease 2019 (COVID-19) infections were identified. Initial efforts were related to contact precautions, hand hygiene, and mask-wearing; however, it was soon evident that a robust global immunization drive was the most effective way to curb disease transmission. In the United States, the first doses of COVID-19 vaccines were rolled out soon after the FDA granted emergency use authorization for the severe acute respiratory syndrome coronavirus 2 (SARS-CoV-2) vaccine. What this also meant was that many of the routine phases that any new drug or vaccine goes through before being released publicly were bypassed. Over the past two years, various side effects and reactions have been seen after COVID-19 vaccine administration, the most common being local injection site events (e.g., pain, redness, swelling) and systemic effects (e.g., fatigue, headaches, myalgias). We report the case of a 64-year-old female who developed bilateral lower extremity numbness and tingling within weeks of receiving the third dose of Moderna SARS-CoV-2 vaccine. The patient underwent extensive testing to ascertain the diagnosis. She had negative autonomic testing and normal nerve conduction study/electromyography (EMG), which did not reveal large fiber neuropathy. Eventually, the patient underwent a skin biopsy, which revealed small fiber neuropathy. This case report highlights the importance of keeping a broad differential for rare side effects, such as small fiber neuropathy, that are currently being seen and reported in the literature.

## Introduction

Some studies report vaccine acceptance rates as low as 56.9% in the general United States (US) population [1]. Vaccine hesitancy usually results in part from lack of trust in vaccination safety and concern related to side effects [2]. Various side effects related to the coronavirus disease 2019 (COVID-19) vaccines have been reported in the past couple of years. Neurological side effects are mainly limited to reports of demyelinating diseases, thrombotic phenomena, and lowering of seizure threshold due to febrile episodes [3]. While small fiber neuropathy is typically linked to diabetes mellitus, amyloidosis, and toxins to name a few predisposing factors, we report the case of an elderly female who developed biopsy-proven small fiber neuropathy after receiving the booster dose of Moderna SARS-CoV-2 vaccine [4].

## Case presentation

A 64-year-old female with a past medical history significant for vertigo, positive antinuclear antibody (ANA), suspected undifferentiated connective tissue disease, hyper IgE syndrome, borderline diabetes mellitus (not on any treatment), and basal cell cancer presented to the emergency department with complaints of one day of chest discomfort, palpitations, and left leg twitching and tingling.

The patient was in her usual state of health up until she developed new symptoms including dull retrosternal pain, palpitations, lightheadedness, and tingling in lower limbs. This was approximately three weeks after receiving the third dose of the Moderna severe acute respiratory syndrome coronavirus 2 (SARS-CoV-2) vaccine. The patient described her symptoms as paroxysmal tingling affecting mainly the feet, L>R. The patient denied back pain, focal weakness, gait changes, or falls. She denied skin rash or loss of muscle bulk. She had presented to the ED due to concern of deep vein thrombosis; however, the Doppler ultrasound that was done was negative. Vital signs were all within normal limits, EKG showed normal sinus rhythm with nonspecific T-wave abnormality and chest x-ray showed no acute pathology. Thyroid-stimulating hormone (TSH) was within normal limits. The patient was asked to remain hydrated over the next few days and to reduce physical activity, and was discharged from the ED. In the following week, the patient followed with Cardiology and had a Holter monitor placed, which only revealed a single event of four beats of ventricular tachycardia on review. 

Her complaints of tingling and numbness in the lower limbs were persistent for which the patient followed up with Neurology. She underwent further workup for neuropathy and palpitations. Autonomic testing was performed, which did not reveal any evidence of parasympathetic cardio-vagal dysfunction, adrenergic dysfunction, postural orthostatic tachycardia syndrome (POTS), small fiber neuropathy, or orthostatic hypotension. Nerve conduction study/electromyography (EMG) did not reveal large fiber neuropathy. The patient eventually underwent a skin biopsy to further evaluate for small fiber neuropathy. Specimens from the distal leg and proximal thigh were consistent with a mild length-dependent neuropathy affecting unmyelinated sensory fibers while specimens from the left ankle revealed occasional small axonal swellings in the epidermis and sub-epidermis. 

## Discussion

Since the start of the COVID-19 pandemic, there has been a global collaborative effort to develop effective vaccinations. Due to the prioritization of early vaccination rollout, the side effects of these vaccinations have not been studied in detail [5].

The sheer magnitude of COVID-19 vaccinations is immense. According to the World Health Organization, 65.7% of the global population has received at least one dose of a COVID-19 vaccine and there are up to six million doses administered daily. Considering the rapid development of COVID-19 vaccinations and the quantity of COVID-19 vaccinations administered, it is imperative that side effects be reported and studied on a global scale [6].

In the past two years, several articles have been published describing different neurological complications following COVID-19 vaccine administration. Finsterer et al. reviewed current literature and found that the four most commonly reported neurological side effects in order of incidence are: headache, Guillain Barre syndrome, venous sinus thrombosis, and transverse myelitis [7]. The fact that these neurological side effects occur after vaccine administration supports a causal relationship between these vaccinations and neurological complications. On the other hand, the relatively small number of reported cases with neurological side effects does show that the risk posed by the vaccine may be minimal [8]. Nevertheless, it is every health provider's ethical duty to report any suspected vaccine side effects. 

We conducted an extensive literature review to find any reported cases of small fiber neuropathy and COVID-19 vaccination. One study reported a 43-year-old male who had a history of neurosarcoidosis and small fiber neuropathy. Three days after his first dose, he had a flare of his underlying neurologic disease. He recovered within two weeks and was able to receive his second dose [9]. A case report published in July 2021 described a 57-year-old woman who presented with symptoms consistent with small fiber neuropathy one week after her second vaccination. She reported resolution in her symptoms in two weeks [10]. Both of the above two cases involved the use of the Pfizer vaccine. We were unable to find any such reports with the Moderna-National Institute of Allergy and Infectious Diseases (NIAID) vaccine. 

To understand the potential relationship between small fiber neuropathy and COVID-19 vaccinations, it is important to appreciate the disease's pathophysiology. It is most likely that autoantibodies target neuronal proteins, which leads to small fiber neuropathy [11]. A study published in April 2022 has shown there is a transient increase in autoantibodies during a COVID-19 infection, which could be due to the activation of T and B cells by recognizing viral epitopes via molecular mimicry [12]. Thus, it can be hypothesized that the vaccination can lead to an increase of autoantibodies, via molecular mimicry, which can result in small fiber neuropathy. However, a study conducted in the United Kingdom has shown there is no significant structural similarity between SARS-CoV-2 genetic or linear protein structure and human peripheral nerve tissue protein structure, making molecular mimicry unlikely. Despite this finding, the authors stated that they can not exclude the possibility that post-translational modification of viral proteins could theoretically result in some form of immunological similarity [13].

The mRNA vaccine, Moderna, has more reports of mild adverse side effects like myalgia and nausea as compared to its counterpart from Pfizer [14]. There is a scarcity of data with regards to serious side effects of the Moderna vaccine. The side effects related to COVID-19 vaccines require long-term monitoring and reporting; however, in the interim, a cautiously optimistic approach toward vaccine administration may be reasonable to adopt. 

## Conclusions

Emergency roll out of COVID-19 vaccines has meant that many of the short- and long-term side effects of the vaccines will only become apparent over years to come. In the meanwhile, it is imperative that even rarely encountered phenomenon be reported so that patients and providers can make more informed and individualized decisions about vaccine safety. Small fiber neuropathy is often difficult to manage due to the inability to ascertain predisposing factors. In the future, if more such cases come up in relation to COVID-19 vaccine administration followed by development of small fiber neuropathy, then prospective studies need to be performed to establish causality. 
